# p53-Mediated Tumor Suppression: DNA-Damage Response and Alternative Mechanisms

**DOI:** 10.3390/cancers11121983

**Published:** 2019-12-09

**Authors:** Consuelo Pitolli, Ying Wang, Eleonora Candi, Yufang Shi, Gerry Melino, Ivano Amelio

**Affiliations:** 1 Department of Experimental Medicine, TOR, University of Rome Tor Vergata, 00133 Roma, Italy; cp674@mrc-tox.cam.ac.uk (C.P.); candi@uniroma2.it (E.C.); gm614@cam.ac.uk (G.M.); 2 MRC Toxicology Unit, University of Cambridge, Cambridge CB2 1QP, UK; 3 CAS Key Laboratory of Tissue Microenvironment and Tumor, Shanghai Institute of Nutrition and Health, Shanghai Institutes for Biological Sciences, University of Chinese Academy of Sciences, Chinese Academy of Sciences, Beijing 100012, China; yingwang@sibs.ac.cn; 4 IDI-IRCCS, Biochemistry Laboratory, 00133 Rome, Italy; 5 Institutes for Translational Medicine, Soochow University, Suzhou 215006, China; yfshi@suda.edu.cn

**Keywords:** cell death, cancer, epigenetics, metabolism

## Abstract

The tumor suppressor p53 regulates different cellular pathways involved in cell survival, DNA repair, apoptosis, and senescence. However, according to an increasing number of studies, the p53-mediated canonical DNA damage response is dispensable for tumor suppression. p53 is involved in mechanisms regulating many other cellular processes, including metabolism, autophagy, and cell migration and invasion, and these pathways might crucially contribute to its tumor suppressor function. In this review we summarize the canonical and non-canonical functions of p53 in an attempt to provide an overview of the potentially crucial aspects related to its tumor suppressor activity.

## 1. “Canonical” p53-Mediated Tumor Suppression

**Figure 1:**
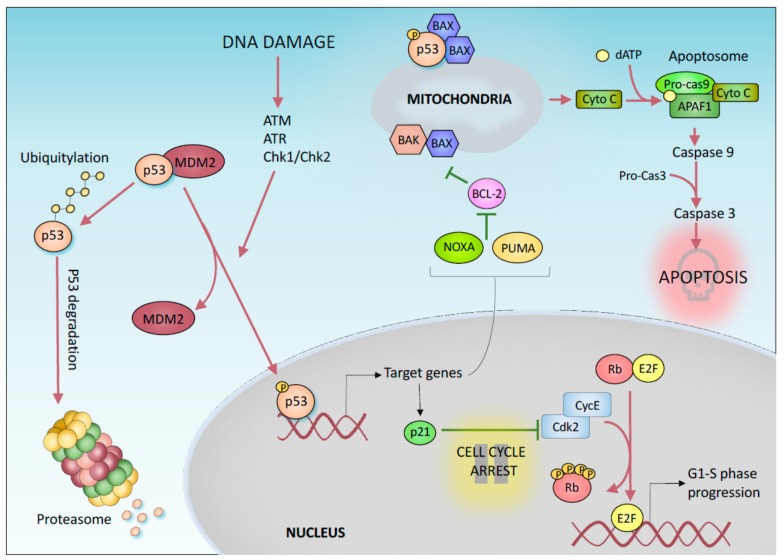
p53-mediated DNA damage response. p53 post-translational modifications determine its stabilization and activation. Downstream transcriptional targets include p21, PUMA, and Noxa, which control cell progression (p21) and cytochrome-C release/apoptosis (PUMA and Noxa). This canonical p53-mediated signalling cascade has for very long been considered the primary mechanism by which p53 prevents tumorigenesis. More recent evidence questions this dogmatic paradigm.

Cancer develops as a result of an uncontrolled cell division. In this context, tumor suppressor proteins play an important role preventing oncogenic transformation by controlling cell growth. Among these genes, the transcription factor p53 has been shown to have a central function. Notably, p53 is the most frequently mutated gene in all human cancers [1]. Mutations in p53 result in a loss of its physiological function, but a gain of novel oncogenic properties has also been suggested [2,3,4]. How does p53 suppress tumorigenesis? The tumor suppressor functions of p53 are mainly associated with its transcriptional activity [5]. Under normal growth conditions, p53 is maintained at a low level by the E3 Ubiquin ligase MDM2, which, in complex with MDM4, mediates p53 proteasomal degradation [6,7,8]. In response to various stress signals (such as DNA damage, oncogene activation, hypoxia, and nutrient depletion), p53 is stabilized and activated by post-translational modifications [9], which include phosphorylation and acetylation [10]. In turn, activated p53 binds to specific DNA sequences in the promoter regions of its target genes, causing several potential tumor suppressive effects (Figure 1) [5,11,12].

The most well-known tumor suppressor function of p53 is based on its ability to promote transient cell cycle arrest, apoptosis, and a permanent form of growth arrest known as senescence [13]. The p53 protein mediates these responses by transcriptionally activating target genes such as p21, which is involved in both permanent and transient cell cycle arrest [14,15,16,17], and proapoptotic BCL2 family members such as Noxa and *BBC3/PUMA* (*BCL2 binding component 3*) [18,19], which mediate apoptosis (Figure 1). In particular, p53 mediated activation of p21 inhibits the activity of cyclin dependent kinases (CDKs) preventing cell cycle transition from G_1_ to S phase. Indeed, CDK inhibition results in retinoblastoma tumor suppressor protein (Rb) hypophosphorylation which impairs E2F-mediated transcription of S-phase promoting genes. In response to a persistent DNA damage, the activation of p53/p21 pathway induces the irreversible arrest of the cell cycle (Figure 1) [20]. These mechanisms are also conserved in the p53 family members p63 and p73, which also perform exclusive developmental functions [21]. In addition to functioning as a transcription factor, transcription-independent mechanisms have been suggested for p53-mediated control of apoptosis. For example, p53 has been reported to directly bind to *BAX* (*BCL2-associated X*, *apoptosis regulator*) in the cytosol, increasing the permeability of the mitochondrial membrane and therefore the efflux of apoptogenic factors, such as Cytochrome C, from the mitochondria (Figure 1). P53 has also been show to control different forms of DNA repair, including mismatch repair, base excision repair, and nucleotide excision repair [22], by directly upregulating DNA repair-associated genes such as *Gadd45a* [23] and by indirectly interacting with repair proteins such as RAD51 [24,25]. Additionally, p53 has been described to play a direct role in the control of replication progression, with several mechanisms which can include or not a direct physical interaction of p53 with the replication fork [26]. Notably, p53 is designated the “guardian of the genome”. Interestingly, unlike p53 knockout mice, mice lacking these canonical p53 effectors (p21, PUMA, and NOXA) are not susceptible to tumor development, suggesting that the ability of p53 to induce apoptosis, cell cycle arrest and/or senescence is unnecessary for its tumor suppressor function [27,28,29]. Thus, the mechanisms that were initially proposed to explain the tumor suppressor property of p53 appear to be reductive. Indeed, p53 also controls many other cellular processes that may contribute to its role in suppressing tumor growth.

## 2. “Non-Canonical” p53-Mediated Tumor Suppression

### 2.1. Regulation of Metabolism

**Figure 2:**
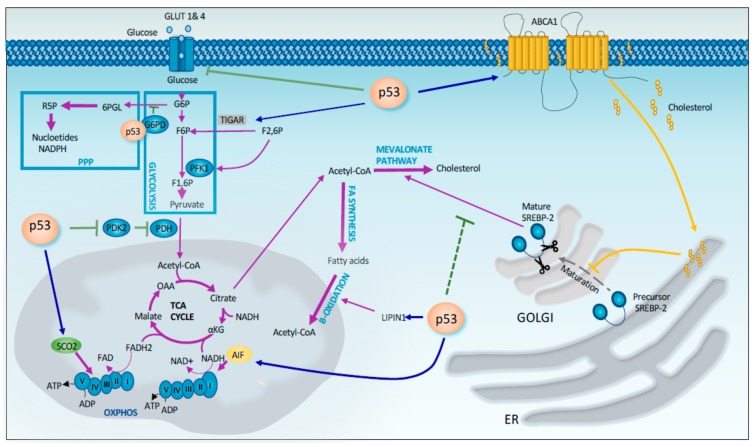
p53 control of cellular metabolism. p53 exerts a stringent control of the cellular metabolism at different level, including efficiency of glycolic pathway, mitochondrial respiration and lipid anabolism/catabolism. p53 directly controls human *Tigar* and *GLUTs* (transcriptionally) and G6PD (by protein-interaction), thus influencing the efficiency of the glycolytic flux and the related anabolic pathways. p53 exerts a control of the mitochondrial activity by influencing *PDK2* and *SCO2* expression. Finally, maturation of SREBP2 and the consequent activation of the cholesterol biosynthesis and fatty acids beta-oxidation are also regulated by p53 activity.

Tumor cells require energy and precursors for macromolecule biosynthesis to sustain their rapid proliferation. Tumor cells undergo metabolic changes to meet these demands. The best-known change in metabolism observed in tumor cells is the Warburg effect. This phenomenon implies that cancer cells prefer to utilize glycolysis rather than the much more efficient oxidative phosphorylation process, even in the presence of sufficient oxygen. Compared to oxidative phosphorylation, glycolysis more rapidly produces ATP in the presence of excess glucose and provide intermediates that are used as precursors for macromolecule biosynthesis through the pentose phosphate pathway (PPP) [30,31], which is crucial for several cancer-related and unrelated processes. In this context, p53 exerts is tumor suppressor function by enhancing mitochondrial respiration and limiting glycolysis and PPP. P53 has been shown to repress the transcription of the transporters GLUT1 and GLUT4, which are involved in glucose uptake in cells [32]. In addition p53 downregulates *GLUT3* gene expression by an indirect mechanism that involves the suppression of IKK-NF-κB pathway (Figure 2) [33]. P53 also reduces glycolysis by inducing the expression of TIGAR (TP53-induced glycolysis regulatory phosphatase), which controls the intracellular level of fructose-2,6-biphosphate, a potent allosteric activator of glycolysis (Figure 2) [34,35]. In addition, p53 promotes the conversion of pyruvate to acetyl-CoA, one substrate of the TCA cycle, by decreasing the expression of PDK2 (pyruvate dehydrogenase kinase 2), which inactivates the pyruvate dehydrogenase complex (Figure 2) [36]. At the same time, p53 negatively regulates the PPP by directly binding and inhibiting G6PD (glucose-6-phosphate dehydrogenase), the first enzyme of this pathway [37]. Thus, p53 reduces the production of NADPH (Dihydronicotinamide-adenine dinucleotide phosphate) and ribose-5-phosphate that are required to sustain tumor growth (Figure 2). On the other hand, p53 enhances mitochondrial respiration by upregulating the expression of target genes such as SCO2 (synthesis of Cytochrome c oxidase 2) and AIF (apoptosis-inducing factor) that are involved in the proper assembly of mitochondrial respiratory complexes (Figure 2) [38,39]. A recent study by the Lowe’s laboratory linked the metabolic effects mediated by p53 deficiency to the changes in control of the cellular epigenome. In particular, the restoration of p53 function in p53^−^ PDAC cells rewires cancer cell metabolism inducing the accumulation of the TCA intermediate, α-ketoglutarate, a metabolite that serves also as a substrate for several chromatin remodeling enzymes. Among these, there are Tet enzymes that promote DNA demethylation through the oxidation of 5-methylcytosine (5-mC) to 5-hydroxymethylcytosine (5-hmC) in an alpha-ketoglutarate dependent manner. Indeed, p53 reactivation in p53^−^ PDAC also induces 5hmC accumulation in a Tet-dependent manner. Interestingly during the progression of human PDAC, the transition from benign to malignant disease is characterized by a 5hmC decrease and in parallel by the loss of wild-type p53. Interestingly, this transition from premalignant lesion to de-differentiated malignant lesions can be prevented by the addition of cell-permeable α-ketoglutarate [40], thus defining a causative link between these two events. These very recent findings keep in line with the previously postulated connection between epigenetic effects of p53 and maintenance of cellular stemness. Activated p53 has been indeed shown to help cells to undergo the developmental lineages commitment through a series of epigenetic changes [41].

In addition to aerobic glycolysis, cancer cells frequently display an increase in fatty acid and cholesterol synthesis. These lipids are used as precursors for the formation of the phospholipid membrane, and thus they are necessary to sustain tumor growth. In this context, p53 plays an important role as a tumor suppressor by promoting oxidation and inhibiting fatty acid synthesis. In particular, p53 induces the expression of Lipin 1 that subsequently coactivates the expression of genes involved in fatty acid oxidation [42]. On the other hand, p53 represses expression of SREBP1c (sterol regulatory element binding protein 1c), a transcription factor involved in inducing the expression of genes associated with fatty acid synthesis [43]. Moreover, p53 interferes with cholesterol synthesis by inhibiting the mevalonate pathway [44]. In particular, p53 controls the activation of SREBP-2, the master transcriptional regulator of this pathway, through the transcriptional induction of the cholesterol transporter gene ABCA1 [44].

In cancer cells metabolic rewiring is often accompanied by increased production of ammonia. In this context, the urea cycle plays an important role in eliminating excess ammonia. p53 transcriptionally represses urea cycle-associated genes, such as CPS1, OTC, and ARG1. The resulting inhibition of urea cycle contributes to p53-mediated tumor suppression. Indeed, tumors generated by p53^−^ HCT116 are larger and show higher levels of urea cycle metabolites compared to p53^+^ HCT116. The simultaneous depletion of CPS1, OTC, and ARG1 impaired tumor growth both in p53 positive and negative tumors. The p53 mediated inhibition of the urea cycle effects tumor growth, increasing ammonia levels. Indeed, ammonia accumulation restrains polyamine biosynthesis, that is required for cell proliferation, by suppressing the translation of ODC1 mRNA, that codify for rate-limiting enzyme of polyamine biosynthesis [45].

### 2.2. p53 and Autophagy

**Figure 3:**
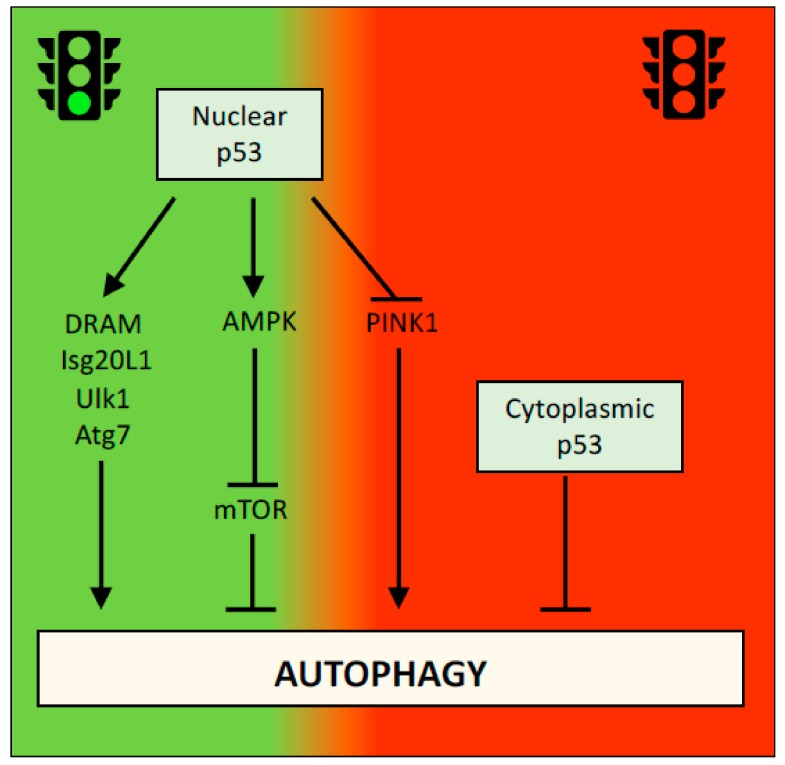
Dual effect of p53 on autophagy. Multiple mechanisms have implicated p53 in the regulation of autophagy with both repressing and promoting effects. Regulation of *DRAM*, *Ulk1*, *Atg7*, and *Isg20L1* results in promotion of autophagy, whereas repression of PINK1 results in promotion of mitophagy. Effect on AMPK/mTOR pathway negatively influences autophagy and similar effect has been ascribed to cytoplasmic functions of p53.

Autophagy is the process by which unnecessary or damaged cellular components are degraded and recycled [46,47]. In presence of enough nutrients, autophagy has an important homeostatic function, ensuring correct protein turnover and organelle quality control. Under unfavorable environmental conditions, such as a lack of nutrients or oxygen, autophagy provides the ATP needed for cell survival. The role of autophagy in cancer is complex. On the one hand, autophagy functions as a tumor suppressor in the early stages of neoplastic transformation to prevent the accumulation of damaged proteins and organelles and reactive oxygen species that induce DNA mutations. On the other hand, the ability of autophagy to support cellular survival in response to stress, such as nutrient or oxygen deprivation, which are frequently observed in growing tumors, might promote the survival of cancer cells [48,49]. P53 has been reported to activate or inhibit autophagy in a context-dependent manner. For example, p53 promotes autophagy by increasing the transcription of several autophagy-associated genes, such as *DRAM*, *Isg20L1*, *Ulk1* and *Atg7* [50,51,52], or by negatively regulating the mTOR pathway [53]. Interestingly, the ability of p53 to inhibit autophagy was initially reported to be independent of its transcriptional activity and exclusively associated with its cytoplasmic localization [54] (Figure 3). However, nuclear p53 was recently shown to inhibit autophagy by downregulating the transcription of PINK1 (PTEN-induced kinase 1), a key protein involved in the mechanism regulating mitophagy [55] (Figure 3). The apparent contradictory findings regarding the effects of p53 on the autophagy flux might however been explained by the alternative and selective effects that p53, and autophagy itself, execute in a context-specific manner.

### 2.3. p53 Opposes EMT and Cell Migration

For metastasis, tumor cells must spread from the primary tumor, invade the surrounding tissues, penetrate the blood vessels and then extravasate to colonize other tissues. This process requires the acquisition of a mesenchymal phenotype, a phenomenon known as the epithelial-to-mesenchymal transition (EMT). Notably, p53 blocks the EMT by inducing the expression of miR-200c and miR34a, which in turn target two important EMT-promoting genes, *Zeb1* and *Snail* [56,57,58,59]. In addition, p53 negatively regulates *Snail* by promoting its degradation by MDM2 [60]. Moreover, p53 suppresses the formation of invadopodia, structures associated with the degradation of the extracellular matrix during cancer invasion, by upregulating the expression of caldesmon and miRNA-143 [61,62]. The p53 protein also restricts cell migration and invasion by modulating the expression of *ROCK1/2* and MRCKα kinases that are the two major effectors of RhoA and CDC42 GTPases, respectively [63]. In particular, in primary human keratinocytes p53 transcriptionally promotes the expression of *Notch* that in turn represses *ROCK1/2* and *MRCKα*. Consistently, in human keratinocytes the suppression of *Notch1* signalling is associated with an increased *ROCK1/2* and *MRCKα* expression and in addition with an increased motility and invasion. Such mobility is suppressed by *ROCK1/2* and *MRCKα* knockdown suggesting these two genes as downstream effectors of p53/Notch1 pathway in the promotion of invasion and migration [64].

The p53 protein also cooperates with other tumor suppressor pathways or represses oncogenic signalling. One of the pathways known to interact with p53 is the Hippo pathway, which is involved in the inhibition of the *YAP* and *TAZ* oncogenes [65]. Moreover, p53 transcriptionally upregulates Ptpn14, a negative regulator of the Yap oncoprotein [66]. On the other hand, p53 suppresses the *c-Myc* oncogene by inducing the expression of miR-145 [67].

### 2.4. Ferroptosis

Ferroptosis is a form of regulated cell death characterized by the iron-dependent accumulation of lipid peroxides [68,69]. GPX4 (glutathione peroxidase 4) is a key regulator of ferroptosis. This enzyme is indirectly inactivated by the inhibition of the cystine/glutamate antiporter system Xc^−^ [70]. This inhibition results in the depletion of cysteine that is used as a precursor for GSH synthesis. In turn, GSH is required for GPX4 activity to catalyse the reduction of lipid peroxides. Thus, the inhibition of system Xc^−^ causes GSH depletion, GPX4 inactivation and the subsequent accumulation of lipid peroxides that initiate ferroptosis [71].

**Figure 4:**
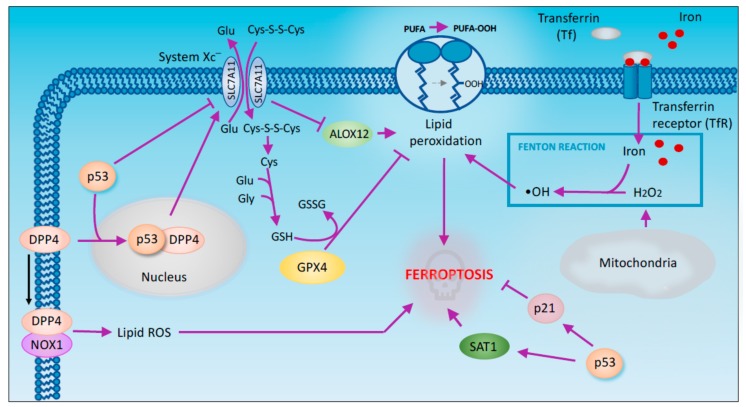
p53 regulates ferroptosis. Iron-dependent lipid peroxidation, more recently named ferroptosis, has been also implicated in the tumor suppression capabilities of p53. Regulation of System Xc^−^ antiport appears to mediate p53 triggered promotion of ferroptotic cell death. Alternative mechanisms implicate p53 in promoting, but also preventing ferroptosis. This include the control of *p21* and *SAT1* expression. The relevance of this cell death modality for p53 mediate tumor suppression opens to novel intriguing perspectives.

A recent in vivo study has highlighted the importance of ferroptosis for p53-mediated tumor suppression. Using p53^3KR^ mice, researchers have shown that p53 inhibits tumor growth partially by repressing the expression of SLC7A11 (a member of cystine/glutamate antiporter) and subsequently inducing ferroptosis [72] (Figure 4). The p53^3KR^ mutant is defective in acetylation and incapable of inducing the expression of the classic p53 target genes but retains the ability to induce ferroptosis. Unlike p53 KO mice, p53^3KR^ mice do not develop tumors. The fact that p53^3KR^ maintains its tumor-suppressing activity suggests that ferroptosis potentially plays crucial roles in the suppression of tumorigenesis [72]. Although this study establishes GPX4 inhibition as the main process associated with p53-dependent ferroptosis, a novel mechanism through which p53 mediates the ferroptotic response has recently been proposed. According to this model, upon ROS-induced stress, p53 indirectly activates the lipoxygenase ALOX12, an enzyme involved in lipid peroxidation, by downregulating SLC7A11 (Figure 4). Consequently, the intracellular levels of lipid peroxides increase, leading to ferroptosis [73]. p53 has been also shown to enhance ferroptosis through the promotion of SAT1, an enzyme acetylating spermidine and spermine using acetyl-coenzyme A [74]. The transcriptional induction of SAT1 by p53 has been shown to promote ROS mediated ferroptosis through the hyperactivation of ALOX15 (arachidonate 15-lipoxygenase). Pharmacologic inhibition of ALOX15 impairs SAT1-mediated ferroptosis, indicating that ALOX15 is a downstream effector of SAT1 [75].

GLS2 is another transcriptional target of p53 family members implicated in ferroptosis [76,77,78]. GLS2 is a mitochondrial glutaminase responsible for glutaminolysis [79]. This process has been shown to be essential for ferroptosis induction upon amino acids starvation. Coherently GLS2 knockdown, impairing glutaminolysis, inhibits serum-dependent ferroptosis in fibroblast [80]. However, GLS2 role in p53-mediated ferroptosis remains to be elucidated.

In facts p53 has been shown to exert a dual effect on ferroptosis. Indeed, p53 also suppresses ferroptosis through the inhibition of DPP4 (dipeptidyl peptidase-4) activity. DPP4 is an enzyme mainly located both in the plasma membrane and in the nucleus. Depending on its subcellular localization DPP4 exerts different activities. In particular in the plasma membrane DPP4 acts as a serine protease, while in the nucleus, it functions as a transcription cofactor [81]. In human colorectal cancer (CRC) p53 forms a complex with DPP4 promoting its redistribution to the nucleus. In absence of p53 DPP4 moves from the nucleus to the cytoplasm where interacts with NADPH oxidase 1 (NOX1) triggering plasma membrane-associated lipid peroxidation that results in ferroptosis Interestingly, DPP4 depletion upregulates SLC7A11 expression in a p53-dependent manner suggesting a role of DPP4 in the control of SLC7A11 expression in TP53^+^ CRC cells [82]. P53 also exerts a pro-survival effect in ferroptosis by inducing CDKN1A/p21 expression. In particular p53-mediated CDKN1A expression delays the onset of ferroptosis. Moreover, compared to p53 depleted cells, wild type p53 cells shows a reduced sensitivity to ferroptosis that requires p53-dependent expression of CDKN1A. However, CDKN1A-mediated cell cycle arrest is not involved in the inhibition of ferroptosis since treatment with CDK4/6 inhibitors is not able to prevent ferroptosis [83].

### 2.5. Maintenance of Genome Stability

Cancer cells are frequently associated with the accumulation of mutations and structural and/or numerical abnormalities in chromosomes. This condition is defined genomic instability. Through its canonical tumor suppressor function, p53 plays a fundamental role in maintaining the integrity of the genome. Importantly, p53 responds to DNA damage, an important cause of genome instability, by inducing cell cycle arrest and promoting DNA damage repair. If DNA damage is not repairable, p53 induces apoptosis. The result of these p53-mediated activities is that the damaged cell does not proliferate, protecting the genome. Alternative mechanisms have also been described in the complex regulation of genome stability by p53. Telomere and centromere maintenance seems to be influenced by p53 through a transcriptional downregulation of gene essentially involved in this mechanism [84].

The p53-mediated regulation of the cell cycle appears to be responsible for preserving the normal cell ploidy. Indeed, tumors derived from transgenic mice expressing the p53 R172P mutant that is incapable of inducing apoptosis but still preserves its ability to arrest the cell cycle are not aneuploid, in contrast to tumors from *p53*-null mice. Moreover, mouse embryonic fibroblasts (MEFs) obtained from *p53^R172P/R172P^* mice also retain a diploid genome, unlike MEFs derived from p53^−^ mice that become tetraploid after a few passages in culture [85]. According to another hypothesis, p53 maintains genomic integrity by restricting mobile elements such as transposons [86,87]. In vivo studies on *Drosophila* and zebrafish models show higher transposon expression in p53 knockout animals than in their wild type counterparts. Consistent with these observations, p53 also mediates retrotransposon de-repression in human and mouse cancers, suggesting that p53 controls transposon mobility [87,88]. However, this hypothesis is based on a correlative observation and formal experimental proof is lacking.

Interestingly, an in vivo study shows that in p53 knockout mice, the thymic lymphomas originate as oligoclonal tumors that evolve dominant clones with time. The exon sequencing of p53 knockout thymic lymphoma samples reveals a very low frequency of point mutations but a huge number of copy number variations (CNVs). Among these mutations, Pten deletion and amplification or overexpression of cyclin Ds and Cdk6, that appear to be common in p53 knockout tumors, have been suggested as driver mutations responsible for tumorigenesis. Interestingly Pten deletion is found in each independent clone of the thymic lymphoma indicating that its loss might occur in early stage of T cells maturation before T-cell receptor addition to their surface. On the contrary Cyclin D Cdk6 overexpression are observed in a small percentage of clones indicating that these mutations might arise after the formation of T-cell receptor, following Pten loss. Thus, thymic lymphomas in p53 knockout mice appear to develop through a particular sequence of genetic alterations that permit the selection of clones of T cells responsible for the initial tumor heterogeneity that finally selects dominant clones [89].

### 2.6. Non-Coding RNA Regulated by p53 in Tumor Suppression

The tumor suppressive functions of p53 are mainly associated with its ability to transcriptionally regulate the expression of many target genes. In addition to protein-coding genes, p53 regulates the expression and biogenesis of many non-coding RNA (ncRNA). One of the best (and firstly) characterized non-coding target gene is the miR-34 family. P53 positively regulates the transcription of miR-34a and miR-34b/c that play an important role in the control of cell proliferation by targeting cyclin E2, cyclin-dependent kinases 4 and 6 (CDK4 and CDK6), and BCL2 [90,91,92]. In addition, p53 directly promotes the transcription of miR-145 that acts as tumor suppressor by targeting the oncogene c-Myc [70]. Other miRNAs induced by p53 are miR-192, miR-194, and miR-215 whose expression is frequently downregulated in several cancers [93]. In particular miR-192 and miR-215 acts as negative regulator of several cell-cycle associated genes [94]. miR-192 upregulation, together with the above-mentioned induction of miR-200 by p53, contributes to downregulate the expression of the EMT-associated genes *ZEB1* and *ZEB2* [58]. p53 can also repress the hypoxia response by inducing miR-107 that targets hypoxia inducible factor-1beta (HIF1beta) [95]. Among miRNAs whose expression is down-regulated by p53 there are miR-224 that inhibits cell proliferation [96], miR-17-92 cluster that promotes hypoxia-mediated cell death [97] (Table 1 for summary). 

p53 has also been shown to regulate miRNAs biogenesis. In particular, p53 interacts with p68, an accessory component of Drosha complex, promoting Drosha-mediated maturation of specific pri-miRNAs. microRNAs whose processing is specifically promoted by p53 include miR-15a, miR-16-1, miR-143, miR-145, miR-199a, and miR-122 that regulate the expression of cell proliferation and stemness associated genes [59].

In addition to miRNAs, p53 regulates also the expression of several long non-coding genes (lncRNA) that contribute to its tumor suppressive function. The p53-responsive lncRNA GUARDIN (RP3-510D11.2-1) plays an important role in the maintenance of genome integrity. In particular GUARDIN sustains the expression of telomeric repeat-binding factor 2 (TRF2), an important factor involved in the protection of chromosome ends, by sequestering its negative regulator miR-23a. In addition, GUARDIN promotes breast cancer 1 (BRCA1) stability and activity by favoring is interaction with BRCA1-associated RING domain protein 1 (BARD1). As a result, GUARDIN inhibition results in the downregulation of TRF2 and decreased activation of DNA repair pathway by BRCA1 [98]. Another p53 lncRNA transcriptional target is NEAT1 that is a constituent of paraspeckle nuclear bodies. NEAT1 has been shown to act as tumor suppressor. NEAT1 is required to suppress transformation in oncogene-expressing fibroblasts and in pancreatic cancer cells and to inhibit pancreatic cancer initiation in vivo [99]. p53 upregulates also PANDA (p21 associated ncRNA DNA damage activated) implicated in cellular senescence. In proliferating cells, PANDA interacts with scaffold-attachment-factor A (SAFA) recruiting polycomb repressive complex (PRC) proteins BMI1-PRC1 and EZH2-PRC2 on senescence-promoting genes repressing their expression. In senescent cells, the expression of the senescence program is allowed by the disruption of SAFA-PANDA-PRC complex, moreover PANDA sequesters the transcription factor NF-YA inhibiting the expression of proliferation-promoting genes coregulated by E2F/NF-YA [100]. Overall an important p53-ncRNA network is emerging in the p53-mediated repression of tumorigenesis.

## 3. Conclusions

Since the discovery of p53, numerous efforts have attempted to decipher its role in cancer as a tumor suppressor. One of the first recognized functions of p53 was its ability to induce cell cycle arrest and apoptosis. For many years, these classic p53 functions were considered to have the major contributions to its tumor suppressor activity. However, the tumor suppressor activity of p53 was recently observed in the absence of these processes. Furthermore, a growing number of studies have revealed roles for p53 in other cellular processes, suggesting that these non-canonical p53 functions may exert a much greater effect on tumor suppression.

Here, we have presented a general overview of the canonical and non-canonical mechanisms by which p53 exerts its oncosuppresive roles. However, despite the extensive literature on the subject, the extent of the contributions of these individual processes and/or whether the function of p53 results from the synergy of several of these molecular pathways remain to be clarified. The flexibility of p53 response dependents on several factors such as cellular specificity and micro-environmental signaling. Depending on the cellular context p53 can exert opposite effects on the same cellular processes. For example, while in breast and lung cancer p53 inhibits glycolysis, in muscle cells p53 was shown to promote this process. Moreover, post-translational modifications of p53 induced in response to various stimuli can determine transcription of different sets of genes leading to distinct biological outputs [101]. Hence, the ability of p53 to protect the cells from extrinsic insults results in different tumor suppressive signaling triggered in a context-specific manner.

## Figures and Tables

**Table 1:** miR up- and down-regulated by p53.

**p53 Upregulated miRs**	**Pathways**	**Targets**
miR-34a/b/c	cell proliferation, EMT	cyclin E2, CDK4, CDK6, BCL2, SNAIL1
miR-145	cell growth	c-Myc
miR-192	EMT	ZEB2
miR-194/miR-215	DNA synthesis, cell cycle	CUL5, LMNB2, CDC7, MAD2L1, BCL2,
miR-200	EMT	ZEB1/2
miR-107	hypoxia response	HIF1β
**p53 Downregulated miRs**	**Pathways**	**Targets**
miR-224	cell proliferation	SMAD4
miR-17-92	apoptosis, cell proliferation	Bim, EGR2, p21
